# The Role of Alternative Medicine in Managing Type 2 Diabetes: A Comprehensive Review

**DOI:** 10.7759/cureus.61965

**Published:** 2024-06-08

**Authors:** Billy McBenedict, Andréa L Orfao, Kang S Goh, Ryan Chun C Yau, Berley Alphonse, Jonatha Machado Lima, Hassan A Ahmed, Gustavo P Ienaco, Elaine Cristina de Souza, Bruno Lima Pessôa, Wilhelmina N Hauwanga, Gabriella Valentim, Magda de Souza Chagas, Ana Abrahão

**Affiliations:** 1 Neurosurgery, Federal Fluminense University, Niterói, BRA; 2 Public Health, Federal Fluminense University, Niterói, BRA; 3 Internal Medicine, Monash University Malaysia, Johor Bahru, MYS; 4 Neurosurgery, Fluminense Federal University, Niterói, BRA; 5 Palliative Care, Brazilian National Cancer Institute, Rio de Janeiro, BRA; 6 Family Medicine, Federal University of the State of Rio de Janeiro, Rio de Janeiro, BRA; 7 Public Health, Fluminense Federal University, Niterói, BRA

**Keywords:** patient education, mind-body therapies, herbal remedies, glycemic control, alternative medicine, complementary medicine, type 2 diabetes

## Abstract

Diabetes, a chronic metabolic disorder marked by elevated blood glucose levels, is increasingly prevalent globally, significantly impacting health-related quality of life. Type 2 diabetes (T2DM), characterized by insulin resistance and inadequate insulin production, presents a substantial public health challenge, necessitating comprehensive management strategies. Conventional treatments, including lifestyle modifications and pharmacotherapy, are essential for glycemic control and preventing complications. However, adherence to these treatments is often limited, highlighting the need for alternative strategies. Complementary and alternative medicine (CAM) offers potential cost-effective and accessible approaches for managing T2DM. Key herbal remedies like cinnamon, fenugreek, and bitter melon, along with dietary supplements like chromium, magnesium, and vanadium, have shown promise in glycemic control. Mind-body therapies, including yoga, tai chi, and meditation, contribute to improved hemoglobin A1c and fasting blood glucose levels. Research supports the integration of CAM with conventional therapies, demonstrating enhanced clinical efficacy and reduced economic burden. However, challenges such as standardization, quality control, and potential risks of herbal medicines need careful consideration. Regulatory frameworks and ethical considerations are essential to ensure patient safety and informed decision-making. Patient education and effective communication between healthcare providers and patients are crucial for integrating CAM into diabetes management. Empowerment-based interventions and collaborative approaches can enhance self-management skills and clinical outcomes. Overall, integrating CAM with conventional treatments offers a holistic approach to managing T2DM, potentially improving patient outcomes and reducing healthcare costs.

## Introduction and background

Diabetes is a prevalent chronic metabolic disorder characterized by elevated blood glucose levels, leading to organ damage over time. Type 2 diabetes (T2DM), associated with insulin resistance or inadequate insulin production, is increasing globally, with urgent public health measures warranted. Diabetes significantly impacts health-related quality of life (HRQoL), with both physical and psychological ramifications exacerbated by complications such as obesity and cardiovascular disease [1]. Longitudinal studies have highlighted the profound effects of diabetes-related complications on HRQoL, emphasizing the need for comprehensive management strategies [1]. Approximately 422 million people worldwide have diabetes, with 1.5 million deaths annually attributed to the disease [2]. In the United States, an estimated 30.3 million Americans have diabetes, with significant proportions undiagnosed, indicating a growing health concern [1]. Adherence to drug treatment aims to control glucose levels and prevent complications, with T2DM presenting a significant public health challenge due to its association with modern lifestyles, necessitating long-term care and generating financial burdens.

Regular physical exercise is recommended as an adjunct to T2DM treatment, offering benefits such as weight loss, reduced cardiovascular risk, and improved glycemic control, potentially decreasing the need for hypoglycemic drugs. Lifestyle intervention strategies have been shown to be highly successful in improving metabolic and functional health among older adults with diabetes, emphasizing their holistic benefits [3]. Structured exercise programs, particularly those combining aerobic and resistance training, have shown superior efficacy in reducing hemoglobin A1c (HbA1c) levels. A reduction in HbA1c is associated with decreased cardiovascular event risks, emphasizing the clinical significance of these interventions. Lifestyle interventions have demonstrated cumulative benefits in improving cardiovascular risk factors [3], thereby reducing morbidity and mortality associated with T2DM. Tobacco cessation is another crucial aspect, as smoking cessation has been linked to reduced mortality from cardiovascular disease. Additionally, dietary modifications, such as adopting the Mediterranean diet or approaches to stop hypertension, are recommended for T2DM [3,4]. These diets, along with weight loss strategies, contribute to improved glycemic control and reduced diabetes incidence. Overall, comprehensive lifestyle interventions offer effective strategies for preventing T2DM in at-risk populations in low- and middle-income countries, although heterogeneity in study outcomes warrants consideration when implementing these interventions.

Pharmacotherapy for T2DM aims to prevent complications and improve quality of life. Metformin is the first-line drug due to its benefits in reducing mortality, promoting weight loss, and improving lipid profiles [5]. Alternative medications like sulfonylureas, dipeptidyl peptidase 4 inhibitors, glucagon-like peptide-1 (GLP-1) receptor agonists, alpha-glucosidase inhibitors, thiazolidinediones, and sodium-glucose transport protein 2 (SGLT2) inhibitors are considered if metformin is unsuitable [5]. Each drug has specific mechanisms and potential side effects, necessitating personalized treatment plans. While metformin remains pivotal, newer agents like SGLT2 inhibitors and GLP-1 receptor agonists offer additional benefits in reducing cardiovascular risks [5]. Innovative approaches to T2DM treatment integrate both pharmacological and non-pharmacological strategies. 

Complementary and alternative medicine offers potential cost-effective and accessible approaches compared to traditional methods. With T2DM presenting a significant global health challenge, marked by insulin resistance and declining pancreatic beta cell function, current strategies relying heavily on expensive medications like metformin may fall short. Therefore, our review aims to provide insights that could lead to reduced healthcare costs and expanded treatment options, ultimately improving outcomes for individuals with this condition.

## Review

**Figure 1:**
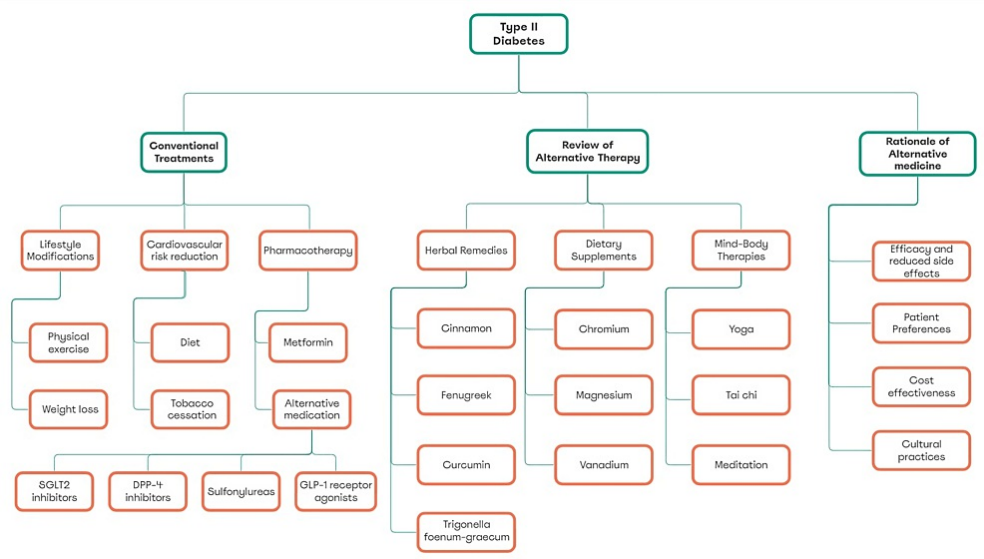
A mind map for the role of alternative medicine in managing type 2 diabetes Generated with Miro Mind Mapping Tool. The mind map was constructed from the articles included in the review [6-56]

This comprehensive review highlights the significant role that complementary and alternative medicine (CAM) can play in the management of T2DM (Figure 1). While conventional treatments such as lifestyle modifications and pharmacotherapy are crucial for glycemic control and preventing complications, the integration of CAM therapies offers additional benefits. Herbal remedies, dietary supplements, and mind-body practices have demonstrated efficacy in improving metabolic outcomes, reducing cardiovascular risks, and enhancing overall quality of life. However, the implementation of CAM therapies must be approached with caution due to challenges in standardization, quality control, and potential risks. This discussion delves into the implications of these findings, emphasizing the need for careful regulation, patient education, and a collaborative approach in diabetes care.

Scope of alternative medicine 

Healthcare professionals globally utilize conventional medicine for managing conditions like hypertension or diabetes mellitus, yet there's a significant prevalence of CAM usage for these diseases. CAM encompasses diverse interventions, practices, and products outside conventional medicine. They define CAM as practices that are not fully integrated into a country's conventional healthcare system [6]. CAM includes therapies like acupuncture, special diets, massage, and medicinal plants [6]. While some CAM therapies are considered complementary, others, like acupuncture and herbal medicine, are standalone treatments [6]. Approximately 30% of T2DM patients utilize CAM, which is influenced by factors such as health literacy and economic status [7]. Certain herbal products like bitter leaf, garlic, and moringa oleifera are popular CAM therapies for diabetes [8]. Moreover, some herbal remedies like Zuo Gui Wan and red raspberry leaves show potential for glycemic control in gestational diabetes [9].

Phloretin targets mechanisms like glucose transport and inflammation in diabetes [10]. Alternative therapies like Korean red ginseng exhibit renoprotective effects against diabetes-induced kidney damage [11]. CAM isn't confined to herbal medicine; mind-body practices like yoga and relaxation techniques also play roles in managing diabetes. Acupressure, a component of traditional Chinese medicine (TCM), demonstrates efficacy in glycemic control, particularly in gestational diabetes [12].

Alternative versus complementary therapies

Complementary medicine represents therapeutic interventions that complement conventional Western medicine, working in conjunction with standard treatments to enhance patient outcomes. In contrast, alternative medicine encompasses practices used independently of mainstream medical approaches, often replacing conventional treatments altogether [13]. However, delineating these realms, complementary, alternative, and integrative medicine, is a multifaceted endeavor. The US National Center for Complementary and Integrative Health defines "complementary" medicine as supplementary to conventional treatments and "alternative" medicine as substitutive for them, with "integrative health" emphasizing the coordinated synthesis of both approaches [14]. Despite attempts to define these categories, boundaries within the domain of CAM remain fluid, challenging traditional taxonomies and necessitating a nuanced exploration of their implications within contemporary healthcare.

Rationale for alternative medicine in diabetes management

TCM therapies, known for their efficacy and reduced side effects compared to pharmaceutical treatments, offer a promising avenue for managing T2DM, often with a lower economic burden [15]. Cultural practices significantly influence diabetes management, with variations in diet and family dynamics playing crucial roles, particularly among Hispanic, Chinese, and Indian populations. Incorporating alternative medicine, such as herbal remedies, aligns with cultural preferences and affordability, shaping patients' treatment choices [16]. Moreover, patients with T2DM frequently turn to online platforms like YouTube for information on CAM, highlighting the importance of accessible health resources in diverse communities [16].

Treating diabetes with plant-derived compounds offers accessibility, cost-effectiveness, and convenience, with evidence showing significant effects on reducing fasting blood glucose (FBG) and postprandial blood sugar levels [17]. Combining TCM with Western medicines like sulfonylurea or metformin enhances clinical efficacy, with TCM treatments often imposing a lower economic burden on patients and payers [17]. Natural agents exert protective and therapeutic effects on diabetes mellitus through various cellular mechanisms, suggesting their potential as adjuvant therapy alongside conventional medications, aiding in insulin dose reduction and HbA1c improvement.

Alternative therapies used in diabetes management

Curcumin, noted for its antioxidant and anti-inflammatory properties, stands out as a promising nutraceutical [18]. *Trigonella foenum-graecum*, or fenugreek, a fiber-rich legume, exhibits blood glucose-lowering effects attributed to its fiber content and bioactive compounds like biguanide-related compounds and 4-hydroxyisoleucine [19]. Fenugreek seed (FS) use leads to significant reductions in FBG and HbA1c, although adverse effects such as flatulence and diarrhea are possible [20]. In addition, *Momordica charantia*, or bitter melon, purported to have insulin-like actions, has shown conflicting results in glucose-lowering efficacy, with recent reviews suggesting a reduction in HbA1c but with limited evidence from randomized controlled trials [21]. Cinnamon, derived from *Cinnamomum cassia* trees, demonstrates insulin-like action attributed to procyanidin polymers, with studies indicating reductions in fasting plasma glucose (FPG) levels [22], but caution is advised when using it alongside other hypoglycemic agents.

Curcumin is potentially useful in managing T2DM, and doses up to 12g per day are deemed safe and tolerable [18]. Dietary supplements like magnesium, chromium, and vanadium have also demonstrated effects on blood glucose levels. Magnesium influences insulin action, and its deficiency correlates with poor glycemic control and complications in T2DM patients [23]. Vanadium, while effective in improving insulin sensitivity, poses challenges due to potential tissue accumulation and toxicity with chronic use [24]. Chromium, a secondary insulin cofactor, shows promise in reducing FBG and HbA1c levels, although findings vary across studies [25].

The feasibility of Iyengar yoga interventions for American adults with T2DM is demonstrated by high adherence (82%) and retention rates (90%) [26]. Mind-body practices, including yoga, meditation, tai chi, acupuncture, and qigong, are integral to CAM. However, numerous adverse effects have been documented linked to acupuncture, including syncope, organ or tissue injury, systemic reactions, infections, and other adverse events [27]. While their mechanisms of action are not fully understood, studies suggest associations between these practices and reductions in HbA1c and FBG levels in individuals with T2DM [28]. Research on yoga demonstrates potential benefits for glycemic control [29], while tai chi has also shown promise in this regard [30]. Some studies have shown positive effects of meditative movements on HbA1c and FBG [31].

While conventional pharmaceutical treatments for T2DM may not always suffice to maintain optimal blood glucose levels or prevent secondary symptoms and medication side effects, TCM therapies have shown promise in clinical studies, offering potential benefits without the adverse effects commonly associated with standard medications [15]. However, the efficacy of CAM in achieving good glycemic control may be limited by certain modifiable factors. Herbal compounds possess hepatoprotective properties and may help to regulate liver metabolism and tissue integrity in diabetes mellitus [32]. Mulberry leaf extract presents a promising adjunctive option for managing postprandial glucose levels in patients with T2DM [33], while cardamom supplementation has been linked to enhanced antioxidant enzyme activity and reduced oxidative stress and inflammation in diabetes mellitus [34].

Honey supplementation over three months alleviated subjective pain scores and symptoms of diabetic neuropathy while improving quality of life in type 2 diabetic patients [35]. Herbal products like Chinese two-herb formula (NF3), Zicao, and Jing Wan Hong ointment, among others, offer the potential for effective treatment of diabetic wounds [36]. Saffron, recognized for its pharmacological properties, including antioxidant effects, shows promise in mitigating diabetes complications by enhancing antioxidant enzyme production and activity while reducing oxidative stress indices [37]. Moderate to low-quality evidence suggests that Chinese herbal medicine may offer beneficial effects on renal function and albuminuria in adults with diabetic kidney disease, surpassing those of conventional treatment alone [38]. The hypoglycemic, antihyperlipidemic, and anti-inflammatory properties of diabetes tea are attributed to its flavonoids, triterpenes, and phytosterol contents [39]. 

In a study, 50 patients with T2DM participated in an eight-week randomized controlled clinical trial to evaluate the effects of FS on serum irisin levels, blood pressure, and liver and kidney function [40]. The intervention group received 5g FS powder three times a day alongside anti-diabetic drugs, while the control group received only anti-diabetic drugs and nutritional consults. Results indicated a significant decrease in FPG and changes in serum alanine aminotransferase and alkaline phosphatase levels in the FS group compared to the control group. Additionally, no notable adverse effects were observed [41].

Various non-herbal products (NHP) have demonstrated efficacy in lowering HbA1c by at least 0.5% in adults with T2DM in randomized controlled trials lasting at least three months. These include Ayurveda polyherbal formulation, *Citrullus colocynthis*, *Coccinia cordifolia*, marine collagen peptides, and others [42]. Additionally, interventions such as Sancaijiangtang and Jinlida have shown promising results in improving cognitive function and glycemic control, respectively, in patients with T2DM [43].

Challenges and limitations of alternative therapies

Herbal medicines, despite being perceived as safe due to their natural origin, can have significant pharmacological activity and interactions with medications, potentially delaying the effectiveness of conventional treatments. Toxicity cases involving CAM therapies in individuals with diabetes highlight potential dangers, including renal failure with chromium picolinate and unsatisfactory outcomes after discontinuing insulin injections for alternative therapies [44]. 

Side effects reported by CAM users include sleepiness, dizziness, sinus issues, and accentuated lowering of blood sugar or pressure [45]. Regarding acupuncture, minor side effects like discomfort and chest pain have been reported, although serious outcomes are rare [46]. Herbal medicine use, while common, is associated with side effects such as headache and nausea, with fenugreek and flaxseed showing potential for gastrointestinal upset [47]. Ginseng, though generally safe, can interact with medications like warfarin, requiring caution [47]. 

Regulatory and ethical considerations

The demand for herbal medicinal products raises concerns about quality control, safety, and efficacy. Inadequate adherence to manufacturing, marketing, and storage protocols can compromise product quality, leading to contamination, variation, and incorrect labeling. Quality control, including substance identification and material purity, is essential for standardization and consistency. 

Regulatory frameworks for traditional and CAM vary globally, with only a fraction of countries having national policies and regulations in place. World Health Organization provides technical guidelines to assist countries, aiming to standardize policies and facilitate information exchange [48]. In the United States, the Food and Drug Administration regulates dietary supplements under the Dietary Supplement Health and Education Act, distinguishing them from drugs and primarily overseeing post-market surveillance [49]. In the European Union, food and medicinal products are regulated separately, with specific standards for each [49]. India's Ministry of Ayurveda, Yoga, and Naturopathy, Unani, Siddha, and Homoeopathy promotes education and research in alternative medicines, with enforcement of good manufacturing practices [48]. 

Legislative controls for medicinal plants vary among countries, leading to differences in licensing, manufacturing, and trading practices [50]. Australia, New Zealand, and Canada regulate natural products under specific laws, ensuring pre-market licensing and assessment [49]. In China, health supplements and medicines undergo pre-market testing and approval, with traditional Chinese medicinal products regulated accordingly [49]. Regulatory approaches reflect this diversity, with some countries establishing robust phytomedicine systems while others treat herbal preparations as food without therapeutic claims [50].

Ethics in clinical medicine necessitates physicians to prioritize patient well-being, prevent harm, and respect patient autonomy. Recommending alternative therapies involves balancing beneficence, non-maleficence, autonomy, and justice. While the principle of beneficence promotes treatments that may benefit patients, many alternative therapies lack robust evidence of safety and efficacy, creating ethical dilemmas. Conversely, the principle of non-maleficence requires physicians to avoid harm, especially pertinent with alternative therapies where risks may be poorly understood. Respecting patient autonomy mandates seeking informed consent, even for uncertain complementary treatments. Justice demands equitable access to therapies, prompting ethical questions on resource allocation and integration into healthcare systems.

Patient education and informed decision-making

A lack of evidence-based information about CAM therapies, alongside insufficient formal training, contributes to uncertainties about efficacy, safety, and potential drug interactions [51]. The widespread use of CAM among cancer patients further underscores these concerns, with a substantial proportion integrating CAM into their treatment regimens [51]. Notably, a survey of Scottish general practitioner offices revealed that a significant percentage of patients were administered herbal medicines known to interact with prescribed conventional drugs [52]. Risks often stem from uncontrolled self-medication with medicinal plants and herbs, which may lead to incorrect dosages or interactions with conventional drugs. Misconceptions about herbal efficacy and safety persist among patients, with many unaware of potential adverse effects [53]. 

While CAM may offer potential benefits, addressing gaps in knowledge and promoting awareness among healthcare providers and patients is crucial. Educational interventions should focus on enhancing understanding, mitigating risks, and promoting informed decision-making regarding CAM use. Nurses and doctors, guided by ethical principles, must advocate for patient safety, especially regarding the use of complementary/alternative therapies. Safety concerns include misleading advertising, inaccurate labeling, and potential interactions with medications [54]. Nurses and doctors must actively assess patients' use of supplements, educate them on potential risks, and advocate for stricter regulations to safeguard patient well-being [54]. Understanding patients' family and social context is vital for ensuring satisfactory care.

Empowerment-based interventions have demonstrated reductions in HbA1c, enhanced self-management skills, and increased knowledge [55]. Collaborative approaches, particularly group-based diabetes self-management education, have shown effectiveness in improving clinical outcomes over six months to two years [55,56]. High follow-up frequency in managed medical centers has also benefited metabolic outcomes, particularly in subgroups with higher baseline HbA1c levels. However, challenges to regular follow-up care and blood glucose monitoring persist, influenced by socioeconomic factors and healthcare access disparities, with urban-rural differences noted in engagement with follow-up care and monitoring [55,56]. Tailored interventions and improved access to diabetes management resources are essential.

Effective communication between healthcare providers and patients is vital for shared decision-making in determining the best clinical approach. Encouraging open discussions about supplement use, respecting patient autonomy, and providing evidence-based information can facilitate informed decisions. Educational interventions and integrating herbal medicine studies into medical school curricula can bridge knowledge gaps and enhance communication regarding herbal medicine. Healthcare providers should initiate discussions about CAM, respecting cultural diversity and beliefs while exploring patients' understanding, preferences, and concerns. Encouraging open-mindedness and providing balanced, evidence-based advice is essential, with consideration given to patients' emotional states and desire for hope and control. Healthcare professionals need adequate training on CAM therapies to ensure safe and effective integration into treatment plans. Respectful communication about NHP and collaboration among healthcare providers further optimize patient care. By considering all criteria for safe CAM use, healthcare providers can promote proper medication and alternative treatment management for chronic diseases.

## Conclusions

The comprehensive management of type 2 diabetes mellitus (T2DM) requires an integrative approach that combines conventional treatments with complementary and alternative medicine (CAM) therapies. While conventional treatments such as lifestyle modifications and pharmacotherapy remain fundamental, the inclusion of CAM offers additional benefits in glycemic control, cost-effectiveness, and alignment with cultural preferences. Herbal remedies, dietary supplements, and mind-body practices have shown promising results in managing T2DM and improving patient outcomes. Despite the potential benefits, challenges such as standardization, quality control, and the risks of herbal medicines necessitate careful regulation and patient education. Effective communication and collaboration between healthcare providers and patients are essential to ensure the safe and informed use of CAM therapies. By integrating CAM with conventional medical practices, healthcare systems can provide a more holistic and patient-centered approach to managing T2DM, ultimately enhancing health-related quality of life and reducing the global burden of diabetes.
